# Case Report: A 42-year-old male with IABP developing multiple organ embolism and intestinal necrosis

**DOI:** 10.3389/fcvm.2024.1335912

**Published:** 2024-02-19

**Authors:** Wanying Yang, Jing Lu, Ting Du, Lulin Sha, Wei Wang, Xin Wang, Qian Gong

**Affiliations:** Department of Cardiovascular Surgery, The First Affiliated Hospital of Anhui Medical University, Hefei, Anhui, China

**Keywords:** acute myocardial infarction, intra-aortic balloon pump (IABP), complication, multiorgan embolism, intestinal necrosis

## Abstract

We report a 42-year-old male patient who was diagnosed with acute myocardial infarction (AMI), and subsequently underwent percutaneous coronary intervention (PCI) for revascularization. The patient was transferred to the cardiac intensive care unit for intra-aortic balloon pump (IABP) due to frequent malignant arrhythmia after PCI. Then the patient experienced the most severe complications of IABP, including multiple organ embolism and intestinal necrosis. This report highlights the rare serious complications of IABP and the challenges encountered in handling this complex case.

## Introduction

Acute myocardial infarction (AMI) is a life-threatening disease that should be timely diagnosed and appropriately treated. Although PCI is an effective intervention to restore coronary artery blood flow, intensive monitoring and care are still required after PCI, and even IABP is needed to increase coronary blood flow, reduce cardiac workload, stabilize coronary circulation and macrocirculation. Although the complications with IABP are relatively rare, the large artery embolism that may occur with IABP is very serious, which is posing significant challenges to the clinical diagnosis and treatment of such patient.

### Case presentation

A 42-year-old male patient weighing 65 kg presented to the hospital with a chief complaint of chest tightness lasting for three hours. Upon admission, the patient's laboratory investigations revealed elevated CKMB levels (501 U/L) and cTnI levels >40 ng/ml. The initial electrocardiogram (ECG) showed QS/Qr morphology in leads II, III, aVF. The patient had elevated blood lipid levels with total cholesterol (TC) (5.42 mmol/L) and triglycerides (TG) measuring 3.22 mmol/L. There was no history of hypertension or diabetes, and no family history of coronary heart disease.

Upon hospital admission (4:40am), the patient received aspirin 0.1 g and low molecular weight heparin calcium 4,100 U for anticoagulation and antiplatelet therapy. An emergency temporary pacemaker was inserted through the right femoral vein. Angiography revealed significant stenosis (60%) in the proximal and middle segments of the left anterior descending artery (LAD), 80% stenosis in the proximal and middle segments of the left circumflex artery (LCX), and acute total occlusion of the proximal and middle segments of the right coronary artery (RCA) with no blood flow (TIMI grade 0). Following a comprehensive evaluation, a stent was successfully deployed to treat the RCA stenosis, achieving complete resolution (0% residual stenosis) on angiography with restoration of TIMI grade 3 blood flow.

During the procedure, the patient experienced multiple episodes of ventricular fibrillation and ventricular tachycardia. Tirofiban was administered (7 ml) to manage the acute coronary syndrome, resulting in a restoration of TIMI grade 3 blood flow on angiography. The PCI procedure started at 5:00am, ended at 7:00am. Subsequently, at 7:50am the patient was transferred to the intensive care unit for further management. To prevent further thrombotic events, the patient received an anticoagulation regimen consisting of aspirin (100 mg, once daily) and ticagrelor (90 mg, twice daily), along with low molecular weight heparin calcium (4,100 U, subcutaneous injection, twice daily) and tirofiban (5 mg/100 ml infusion, twice daily).

Despite the initial interventions, the patient continued to experience intermittent episodes of ventricular tachycardia and frequent premature ventricular contractions. As a result, at 15:50pm on the day the patient entered ICU, the IABP support was initiated to provide auxiliary circulatory support and stabilize the patient's condition.

On the 8th day of hospitalization (PCI and IABP), the patient developed a dull pain in the left upper abdomen, accompanied by abdominal distension, with alanine aminotransferase (ALT) began to increasing. The pain subsequently shifted to the right lower abdomen. There were no nausea or vomiting and tenderness or rebound tenderness.

Laboratory indicators on the 8th day revealed an elevated D-dimer level (>20 µg/ml), with cardiac biomarkers CKMB measuring 26 U/L and cTnI level at 2.156 ng/ml (continued its downward trend), and ALT started to rise (154 U/L). The intestinal sounds lightly weak, 2–3 times per minute. The levels of blood amylase and lipase were within normal limits on admission, on the 8th day and 9th day. On the 9th day serum creatinine began its upward trend (131 mmol/L), and an ECG showed ST segment depression and T wave even inversion in leads III, AVF, and V5∼V6. Abdominal vascular ultrasound did not show any abnormalities, while a CT scan demonstrated gas accumulation in the upper gastrointestinal tract and elevated troponin levels. From these above findings, our group still focused on the “heart pump” problem, and the patient maybe the suspect of perioperative recurrent acute myocardial infarction, so IABP was kept, and low molecular weight heparin calcium was replaced with a heparin pump to intensify anticoagulation therapy.

From the 8th to the 10th day, we still focused on dealing with the heart issue. On the 10th day, we even administered intravenous analgesics (remifentanil 8 mg/24 h) to the patient in an attempt to alleviate his pain. However, cardiac injury biomarkers CKMB and cTnI levels continued to trend lower which did not support the diagnosis of perioperative recurrent acute myocardial infarction. The patient still had abdominal pain and began to experience bloating, though bowel movement every day. On the 10th day, we began monitoring the patient's abdominal circumference (93 cm) and intra-abdominal pressure (5.5 cmH2O).

**Figure 1:**
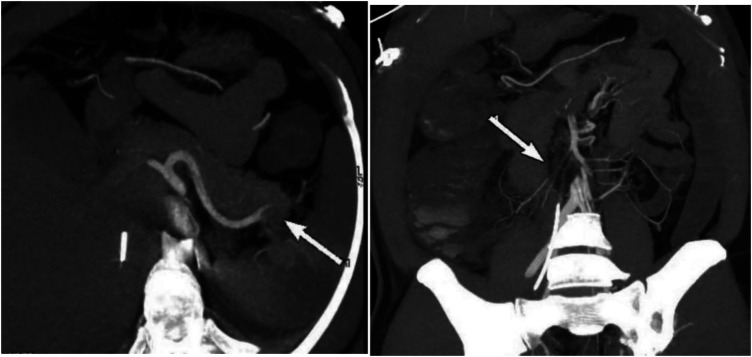
Preoperative CTA showed an acute completely embolic occlusion of arteria mesenterica superior and splenic artery (arrow).

On the 11th day of hospitalization (PCI and IABP) the patient's abdominal pain did not improve, and urine output significantly decreased. The patient's abdominal circumference (95 cm) and intra-abdominal pressure (7.5 cmH2O) were both increasing on the 12th day, ALT reached its peak (154 U/L), and the levels of blood lipase and amylase were a slight increase and lipase exceeded the normal upper limit, so abdominal vascular computed tomography angiography (CTA) was performed and revealed multiple thromboses involving the celiac trunk, upper and lower mesenteric arteries, splenic artery, bilateral renal arteries, left inferior gluteal artery, as well as branches of the proper hepatic artery. These thrombotic events led to secondary infarction of the spleen and bilateral kidneys. Additionally, gas accumulation in the intestinal wall of the ascending colon and suspected ischemic necrosis of the distal ileal intestinal wall were noted, indicating ischemic bowel involvement (Figure 1).

Following the removal of the temporary pacemaker and IABP immediately once CTA detected thrombi in several branches of the abdominal aorta on the 12th day of hospitalization (PCI and IABP), emergency celiac arteriography was promptly performed. Urokinase thrombolysis and interventional thrombectomy were performed at the main renal arteries, as well as the gastroduodenal, left hepatic, and right hepatic arteries. The first thrombolysis procedure completed. On the 14th day, the intestinal sounds remained weak, one time per minute the patient's abdominal circumference (97 cm) and intra-abdominal pressure (7.3 cmH2O) was stable. Subsequent follow-up examinations revealed a significant reduction in thrombus burden.

Continuous renal replacement therapy (CRRT) was initiated immediately after thrombolysis, and the highest creatinine level is 600 mmol/L before CRRT, with concurrent continuous infusion of heparin to maintain the activated partial thromboplastin time (APTT) between 45 and 60 s. The patient's abdominal pain and urine output improved. However, abdominal distension persisted, and the patient experienced difficulty with defecation and flatus. On the 15th day, the intestinal sounds remained weak, 1–2 times per minute. The patient's abdominal circumference (95 cm) slightly decreased, however intra-abdominal pressure reached a maximum of 10.5 cmH2O.

**Figure 2:**
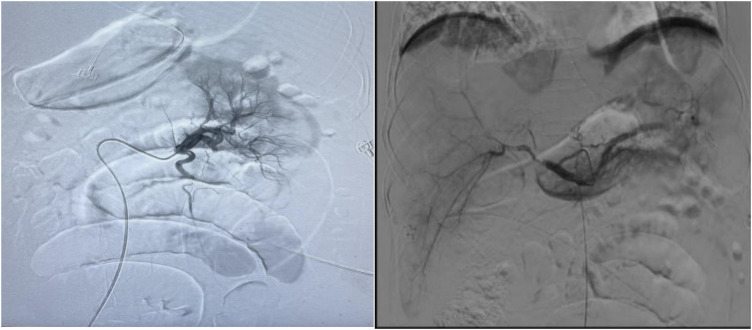
Urokinase dissolves thrombus at the opening of both renal arteries and superior mesenteric arteries, while loach guide wire pulls thrombus at the gastroduodenal artery, left hepatic artery, right hepatic artery, and superior mesenteric artery.

On the 4th day after the first thrombolysis procedure, celiac arteriography was performed again. The blood flow perfusion of the left and right hepatic arteries and bilateral renal arteries was significantly improved. Further thrombolysis with urokinase was administered to the left renal artery and the main trunk of the superior mesenteric artery. The second thrombolysis procedure was completed (Figure 2).

**Figure 3:**
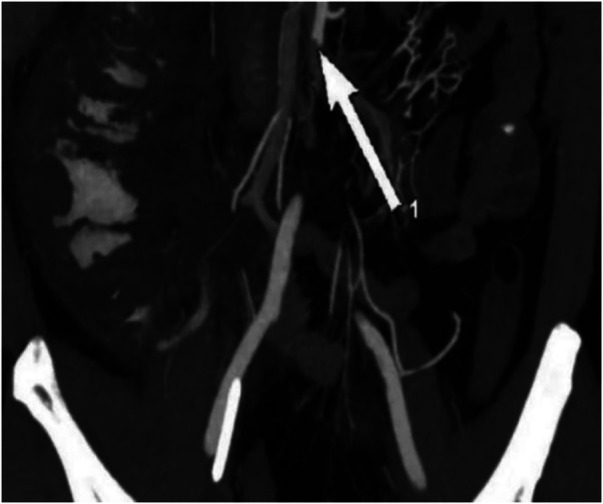
Abdominal artery embolism and signs of partial colon necrosis with inflation (arrow).

After the second procedure, the patient’s bowel movements and ventilation improved, along with an improvement in urine output. However, the patient continued to experience abdominal pain and abdominal distension without any intestinal sound. The total bilirubin level reached its highest level of 46.81 mmol/L. The intra-abdominal pressure gradually decreased to 5.5 cmH2O on the 17th day and quickly increased to 9.2 cmH2O on the 20th day. The patient has feces every day, ranging from 200 ml to 600 ml per day, but on the 20th day, he began to have bloody stool. Additionally, symptoms of peritonitis and signs of shock were observed. The second CTA suggested signs of intestinal necrosis, which needed an urgent surgical intervention to evaluate the extent of the necrosis and potentially remove any nonviable tissue. (Figure 3).

On the 21st day of hospitalization (PCI and IABP), the patient underwent an emergency surgery involving partial resection of the small intestine and colon, an ileostomy, release of intestinal adhesions, and peritoneal irrigation and drainage. Approximately 1.2 meters of the jejunum were removed during the procedure. The patient gradually recovered after the emergency surgery.

Six months after being discharged, the patient underwent a stoma reduction procedure and was experiencing a favorable recovery. The patient exhibits good liver function, and his kidney function prompted the creatinine value was in the range of 120 to 140 mmol/L, and his urine volume is normal.

A multidisciplinary consultation suggested to investigate the possibility of thrombophilia (a tendency to develop abnormal blood clots). So various examinations were performed including tests for antinuclear antibodies (ANA), anticardiolipin antibodies (ACL), anticoagulant lipid syndrome, tumor markers, paroxysmal nocturnal hemoglobinuria (PNH), protein C and S deficiencies, antithrombin III levels, endogenous coagulation factor VIII levels, homocysteine levels, and other related tests. The results indicated that the patient had elevated levels of anticardiolipin antibody IgG (9.62 GPLU/ml) and endogenous coagulation factor VIII (155.1%). The tests for Lupus anticoagulant (LA1/LA2) were within a normal level. Gene detection resulted in negative.

### Outcome and follow-up

After the stoma reduction procedure, the patient received a supportive care including anticoagulation and antiplatelet therapy. A regular and close follow-up of the patient confirmed the satisfactory recovery. However, his serum creatinine value was 130 mmol/L or so.

## Discussion

IABP (Intra-Aortic Balloon Pump) is the most commonly used mechanical circulatory support device, which is primarily utilized to treat cardiogenic shock. It can reduce the afterload of the heart and increase coronary artery perfusion (1).

The balloon of IABP rapidly inflates during the diastolic phase of the heart, causing an increase in diastolic pressure in the aorta, which can increase aortic diastolic pressure by 30%–70% compared with before the assistance. The balloon rapidly deflates before the systolic phase of the heart, resulting in a 5%–30% decrease in aortic pressure (the end diastolic aortic pressure), a decrease in left ventricular afterload, and an increase in cardiac output of 0.5–1.0 L/min, with a maximum increase of about 15%. These significantly improve the balance of myocardial oxygen supply and demand, and also improve coronary artery blood flow under the endocardium (2). The complications of IABP mainly include limb ischemia, severe bleeding, balloon rupture, and the death directly related to the failure of IABP implantation, with an incidence rate of 2.6%. Women, advanced age, and peripheral vascular disease are high-risk factors (3). The most significant drawback of IABP is the occurrence of ischemic vascular complications, primarily resulting from mechanical, thrombotic, or embolic occlusion of aortic branches (4, 5). Previous studies have indicated that lower extremity ischemic complications are more prevalent in patients with peripheral vascular disease, with an incidence ranging from 2.6% to 11.7% (6, 7). Because patients with cardiogenic shock often exhibit insufficient peripheral tissue and organ perfusion, and acute myocardial infarction patients often have peripheral vascular disease. Therefore, patients with acute myocardial infarction are more prone to lower limb ischemia during using IABP.

However, in our case, the patient experienced rapid failure of multiple organs caused by abdominal multivascular emboli at the 8th day after IABP. The initial symptom was abdominal pain, which was accompanied by increased levels of D-dimer and creatinine. An abdominal CT examination did not reveal any significant abnormalities, but myocardial enzymes were elevated. Considering the possibility of recurrent AMI in the perioperative period, a more aggressive anticoagulation program was prescribed, and IABP was kept that was a high risk factor for thrombosis in abdominal aortic branches. Then we found the increase in liver injury indicators on the 8th day and focused on cardiogenic factors, not investigated other causes, especially fatal thrombosis. Because of our neglect, the patient's condition gradually worsened, with increasing abdominal distension and persistent high D-dimer values, suggesting the possibility of acute superior mesenteric artery embolism (ASMAE). This diagnosis was confirmed by emergency abdominal CTA. Although thrombus removal and thrombolysis were performed, intestinal necrosis was not avoided due to ischemia-reperfusion injury.

ASMAE is a rare acute abdominal disease with an insidious onset and atypical early-stage symptoms. Information regarding ASMAE is scarce, and the rate of misdiagnosis and missed diagnosis is extremely high. It is often associated with conditions such as aortic dissection, pancreatitis, and cholelithiasis. Upper gastrointestinal bleeding and other diseases can be mistaken for ASMAE. If an early diagnosis is not made, the condition can progress to extensive intestinal necrosis, septic shock, and multiple organ failure, resulting in an extremely high fatality rate. Intestinal ischemia is often severe and can lead to intestinal necrosis, highlighting the importance of early diagnosis and timely and effective treatment (8).

Diagnostically, D-dimer is a plasma marker that reflects the state of hypercoagulability or secondary hyperfibrinolysis. It has high sensitivity but low specificity. Normal D-dimer levels can help rule out mesenteric upper artery thrombotic occlusive lesions (9). Abdominal vascular color Doppler ultrasound is convenient, but cannot detect early abdominal thrombosis, and early abdominal vascular CTA is more effective. Abdominal contrast-enhanced CT or CTA allows for the observation of mesenteric vessels and their branches, assessment of bowel ischemia, evaluation of the abdominal cavity condition, and differentiation from other abdominal diseases. The patient's abdominal vascular ultrasound and CT plain scan failed to identify the formation of arterial embolism very early, which also delayed the optimal timing of the thrombolysis and thrombectomy treatment. Therefore, we advocate that clinical physicians should be highly vigilant of ASMAE when IABP patients experience abdominal pain symptoms, and complete CTA or angiography as soon as possible.

In this case study, the patient presented with a large area of abdominal vascular embolism. In addition to intestinal necrosis caused by embolization of the superior mesenteric artery, the arteries of the kidney and spleen were severely blocked, which leaded to acute kidney injury. Thrombolytic therapy surgery preserved the kidney function. During the two-year follow-up, the patient's serum creatinine value was approximately 130 mmol/L, and his urine output was normal.

This highlights the importance of recognizing early signs of ASMAE, such as abdominal pain, distension, or obstruction symptoms, in patients with a history of cardiac issues and arteriosclerosis. Especially, clinical doctors should attach great importance to the rare complication and avoid secondary organ damage caused by ischemia-reperfusion. The high awareness of clinical doctors regarding rare diseases should help early diagnosis. Once diagnosed, the right choice of treatment plan is crucial, and the rare complication requires rapid diagnosis, resuscitation, and early revascularization (10).

According to the 2016 ESTES (European Society of Trauma and Emergency Surgery) guidelines for acute mesenteric ischemia, endovascular treatment can be considered in cases where immediate surgical intervention is not required (11). The goal of surgical treatment is to restore blood flow to the superior mesenteric artery and remove any necrotic bowel tissue. The timing of surgery plays a key role in reducing the fatality rate. When conservative and interventional treatments fail to control the condition, exploratory laparotomy should be performed.

In the current case study, the patient primarily experienced abdominal pain, bloating, and incomplete intestinal obstruction. After two interventional treatments, CTA revealed intestinal necrosis, necessitating the removal of a part of the small intestine and fistula creation. Is it possible to avoid intestinal necrosis caused by ischemia-reperfusion injury in the patient and to avoid the intestinal resection surgery if the CTA time is advanced, as there are four days from the onset of abdominal pain and DD elevation significantly to the intervention of thrombolysis and thrombectomy.

The patient's height was 170 cm, and weight was 65 kg. The 40 cc balloon (Arrow tube) used was appropriate and functioned well. The position of the catheter tip was regularly examined to ensure proper placement, and heparin was flushed every half hour at a dose of 130 u, and APPT was in the scope of 38.4–41.1 s before the occurence of dull pain in the left upper abdomen.

Considering the reasons for the development of ASMAE, several factors can be considered:

1. The patient had AMI and experienced repeated ventricular fibrillation after defibrillation. During this process, microemboli may have become dislodged, leading to an embolic cascade reaction within the abdominal blood vessels, further exacerbating the process of multivessel embolization.
2. Vasogenic emboli, such as dislodgement of atherosclerotic plaques, thrombus dislodgement from an aneurysm wall, or left ventricular thrombus caused by intervention with catheters, can contribute to the development of ASMAE (12).
3. A sudden decrease in intestinal blood flow, typically resulting from systemic circulation disorders such as congestive heart failure, myocardial infarction, arrhythmia, shock, post-traumatic hemorrhage, or excessive use of vasoconstrictor drugs, can also contribute to the occurrence of ASMAE (13).
4. IABP was implanted in the aorta beyond one week.

These factors, combined with the patient's underlying cardiac issues and arteriosclerosis, likely contributed to the development of ASMAE in this case.

Studies have shown that among young MI patients with one traditional risk factor for coronary heart disease, the incidence of thrombophilia is around 15% (14). The elevated levels of anticardiolipin antibody IgG and endogenous coagulation factor VIII suggest that the patient may have an underlying thrombophilic tendency, which could have contributed to the development of ASMAE in this case, combined with the use of IABP, may have aggravated the condition.

While serious complications after aortic balloon counterpulsation following PCI treatment for AMI are rare, they can result in very poor prognosis. In cases where complications related to superior mesenteric artery thrombosis arise, prompt superior mesenteric arteriography should be conducted. Early diagnosis, timely intervention, removing the trigger, anticoagulation strategy, the thrombolysis and thrombectomy treatment plan as early as possible or surgical treatment to prevent intestinal necrosis are crucial for improving the prognosis.

Overall, a comprehensive understanding of the underlying factors and prompt management are essential to address the complications associated with IABP and to improve patient outcomes.

## Data Availability

The original contributions presented in the study are included in the article/Supplementary Material, further inquiries can be directed to the corresponding author.
